# Support vector machines-based identification of alternative splicing in *Arabidopsis thaliana *from whole-genome tiling arrays

**DOI:** 10.1186/1471-2105-12-55

**Published:** 2011-02-16

**Authors:** Johannes Eichner, Georg Zeller, Sascha Laubinger, Gunnar Rätsch

**Affiliations:** 1 Friedrich Miescher Laboratory, Max Planck Society, Spemannstr. 39, 72076 Tübingen, Germany; 2 Max Planck Institute for Developmental Biology, Spemannstr. 35, 72076 Tübingen, Germany; 3 Centre for Bioinformatics, University of Tübingen, Sand 1, 72076 Tübingen, Germany; 4 European Molecular Biology Laboratory, Meyerhofstr. 1, 69117 Heidelberg, Germany; 5 Center for Plant Molecular Biology, University of Tübingen, Auf der Morgenstelle, Germany

## Abstract

**Background:**

Alternative splicing (AS) is a process which generates several distinct mRNA isoforms from the same gene by splicing different portions out of the precursor transcript. Due to the (patho-)physiological importance of AS, a complete inventory of AS is of great interest. While this is in reach for human and mammalian model organisms, our knowledge of AS in plants has remained more incomplete. Experimental approaches for monitoring AS are either based on transcript sequencing or rely on hybridization to DNA microarrays. Among the microarray platforms facilitating the discovery of AS events, tiling arrays are well-suited for identifying intron retention, the most prevalent type of AS in plants. However, analyzing tiling array data is challenging, because of high noise levels and limited probe coverage.

**Results:**

In this work, we present a novel method to detect intron retentions (IR) and exon skips (ES) from tiling arrays. While statistical tests have typically been proposed for this purpose, our method instead utilizes support vector machines (SVMs) which are appreciated for their accuracy and robustness to noise. Existing EST and cDNA sequences served for supervised training and evaluation. Analyzing a large collection of publicly available microarray and sequence data for the model plant *A. thaliana*, we demonstrated that our method is more accurate than existing approaches. The method was applied in a genome-wide screen which resulted in the discovery of 1,355 IR events. A comparison of these IR events to the TAIR annotation and a large set of short-read RNA-seq data showed that 830 of the predicted IR events are novel and that 525 events (39%) overlap with either the TAIR annotation or the IR events inferred from the RNA-seq data.

**Conclusions:**

The method developed in this work expands the scarce repertoire of analysis tools for the identification of alternative mRNA splicing from whole-genome tiling arrays. Our predictions are highly enriched with known AS events and complement the *A. thaliana *genome annotation with respect to AS. Since all predicted AS events can be precisely attributed to experimental conditions, our work provides a basis for follow-up studies focused on the elucidation of the regulatory mechanisms underlying tissue-specific and stress-dependent AS in plants.

## Background

### Alternative splicing

Alternative splicing (AS) is an important mechanism implicated in eukaryotic gene expression, whereby exon segments of precursor-mRNA transcripts are joined together in different arrangements. In contrast to constitutive splicing, where all exons of a gene are joined together in a single fixed composition, AS is thus a mechanism which generates distinct mature mRNA transcripts from the same gene by variable use of splice sites. Many different types of AS events are known so far. The most common types are exon skipping, intron retention and the alternative usage of 5' or 3' splice sites. Exon skips have been shown to be the most prevalent type in mammals, whereas intron retentions account for most AS events in plant systems, such as *A. thaliana *[1-3]. Accumulating evidence suggests that in many instances the generation of alternative isoforms of a gene is not just transcriptional noise, but a specifically regulated process of physiological importance, as it, for instance, substantially contributes to the structural and functional diversification of cell types [4]. Consistent with this view, several studies of AS in different organisms reported that AS events may undergo differential regulation between tissues, i.e., the ratios of alternative transcript isoforms were observed to vary across tissues [5,6]. This suggests that tissue-specific differential splicing plays a major role in the evolution of specialized cell and tissue types [7,8]. Moreover, misregulation of AS may give rise to pathophysiological processes and has been associated with human diseases, such as cancer [9], cystic fibrosis [10], and many others [11]. Since AS has been extensively studied in mammals, but to a notably lesser extent in plants, we focused on the well-established model organism *A. thaliana *for investigating the regulation and prevalence of AS in plants. Environmental stresses have been found to impact AS in plants, and novel transcript isoforms appearing under biotic or abiotic stresses have been reported [3,12-14]. Stress-induced AS is supposed to be mediated by altered levels, localization, or phosphorylation status of splicing factors [3]. Consequently, levels of mRNA isoforms change or new splice variants appear. This regulatory mechanism enables sessile plants to adapt their transcriptome in response to substantial environmental changes. Cold and heat stress, for instance, have been shown to result in altered splicing of SR protein pre-mRNAs [15,16], which are known to act as splicing factors that in turn affect alternative splicing of pre-mRNAs at a more global level. However, the whole complexity of the interaction network underlying stress-regulated AS events still remains to be elucidated by experimental and computational in-depth studies of AS in plants. Large-scale studies of AS are either based on sequencing mRNA transcripts or on the inference of AS events from splicing sensitive microarrays. Sequencing approaches are typically based on a mapping of expressed transcripts (e.g. ESTs, cDNAs or deep sequencing reads) to the genomic sequence of the studied organism using a spliced alignment algorithm, such as BLAT [17]. Array-based approaches utilize microarray platforms which are purpose-built for the highly parallel quantitative profiling of alternative transcript isoforms on a genome-wide level. Unless very deep sequencing of transcriptomes becomes a routine and affordable, splicing-sensitive microarrays are still a viable alternative for detecting and profiling transcript isoforms[18].

### Splicing-sensitive microarray platforms

Since microarrays commonly used for gene expression profiling do not generally allow to accurately detect AS events, special array platforms have been developed for the characterization of transcript isoform variation. Most of them can be assigned to one of the following categories: tiling arrays, exon arrays and exon junction arrays [19], each of which comes with its own advantages and limitations. Exon or exon junction arrays as well as tiling arrays enable *de novo *discovery of new transcript isoforms. One distinguishing feature of tiling arrays is that they are unbiased, as the tiling probes are designed independently of genome annotations. To measure the expression of individual isoforms relative to the overall expression of a gene, microarrays comprising both exon body as well as isoform-specific exon junction probes, have been widely used [6,8]. The design of these arrays is often focused on a particular gene set of interest. In contrast to that, tiling arrays typically interrogate the whole non-repetitive portion of a genome with equally spaced probes, a design which is particularly suited for compact genomes, such as that of *A. thaliana*. Moreover, in contrast to exon arrays, which are mostly limited to monitoring exon skips and alternative 5' and 3' splicing owing to their probe design, tiling probes complementary to intronic regions enable the discovery of intron retention events, which account for more than 50% of the known AS events in *A. thaliana *[2]. Theoretically, all types of AS are detectable from tiling arrays, but in practice, probe density is a limiting factor. For instance the detection of alternative 3' or 5' splice site selection is very difficult with the Arabidopsis Tiling Array 1.0R by Affymetrix, because a substantial fraction of these events can only be detected from changes in a single probe set (44% of the ones annotated in TAIR7).

### Methods for the analysis of splicing microarray data

Previous array-based approaches aiming at global analysis of AS in diverse organisms are mostly unsupervised and typically based on statistical testing. The widely used method MIDAS http://www.affymetrix.com/support/technical/whitepapers/exon_alt_transcript_analysis_whitepaper.pdf, which is part of the freely available R/Bioconductor package exonmap, [20] is based on the assumption that the signal level of an exon relative to the overall gene signal level is constant over samples for constitutively spliced exons. However, in the presence of AS significant differences in the logarithmized ratio of normalized exon and gene levels, which is referred to as splicing index, can be detected over samples using an ANOVA test. MADS is an algorithmically similar approach which is also based on splicing indices [21]. The main difference to MIDAS, which computes p-values on probeset-level, is that MADS performs the statistical test for the detection of differential splicing indices across samples on the probe-level and performs the summarization over probes afterwards. Purdom *et al*. developed another unsupervised method for the detection of AS from exon arrays, which is called FIRMA [22]. FIRMA extends the very popular robust multi-chip analysis (RMA) model. AS events are in essence inferred from high residuals of individual exon probe sets in the base RMA gene-expression model, since these indicate a high deviation of the observed from the expected signal level of an exon. All of the above-mentioned methods are based on the assumption that the expression level of single exons is equal to the overall gene expression level in the absence of alternative splicing, and consequently exon skips are inferred based on deviations from this behavior. However, a recent study reported that in practice a technical bias of exon array platforms leads to a dramatic overestimation of AS in the presence of differential gene expression. To correct for this bias, the authors developed the method COSIE, which adjusts splicing indices using a non-linear model that incorporates probeset-specific response characteristics and saturation effects [23]. All of these computational methods were developed for the analysis of data from exon (junction) arrays, and in practice they cannot easily be applied to tiling array platforms. Conceptually, however, many of their modeling approaches are applicable to tiling arrays as well. Most notably, the concept of relating the hybridization pattern of an exon or intron, for which AS is to be tested, to those of surrounding exons and introns in the same gene can be transferred to tiling arrays. One of the first approaches for tiling array-based inference of AS was developed by Ner-Gaon *et al*. [24]. It discovers retained introns based on untypically high hybridization signals. Specifically, hybridization intensities measured for individual introns of a gene are related to each other and to the mean exonic signal level by means of a one-way ANOVA test. Building on the key concept common to many of the above-mentioned methods that the hybridization pattern of a putatively skipped exon is expected to be dissimilar to those of surrounding exons (and likewise that retained introns deviate from the typical intron hybridization pattern), we developed a method for tiling array data which uses a new principle of inference. Instead of employing a statistical test, our method is based on supervised learning, and specifically makes use of well-studied Support Vector Machine (SVM) classifiers (see [25,26] and references therein). In contrast to the unsupervised testing approaches, our method is trained on known AS events obtained from EST/cDNA databases or genome annotations. It proceeds in two steps: First, we predict single-sample confidence scores measuring the inclusion level of an exon or intron in the mature mRNA transcripts of a gene. In a second step, we integrate these confidences across samples to predict all-sample confidence scores for alternative splicing.

## Results

We focused on two types of alternative splicing (AS) events, namely exon skips (ES) and intron retentions (IR), which can be detected sufficiently accurately with the given tiling array resolution.

A supervised machine learning approach for the identification of IR and ES requires a set of "labeled data", i.e., exons and introns which are known to be subject to AS or not. Such labeled data are needed for the training of classifiers, and provide a reference for the evaluation of the prediction accuracy and comparisons of different detection methods. Owing to extensive previous work, several thousand AS events have been annotated and confirmed by EST and cDNA sequences [27,28]. As the SVM classifiers had to distinguish alternative from constitutive splicing, we also collected examples which are unlikely to be alternatively spliced. However, despite the existence of several AS databases and continuous improvements of gene annotations [29,2,27], it remains a challenging task to assess for exons and introns which are not annotated as alternatively spliced, whether alternative isoforms may exist, but have not yet been sequenced. Arguably, exons or introns which are confirmed by many sequenced transcripts, none of which reveals an alternative isoform, are the best candidates for true constitutive splicing events [30].

### AS confirmed by EST and cDNA sequences

After aligning ESTs and cDNAs to the genome and inferring exon-intron structures (see Methods), we obtained confirmation counts for each exon and intron as the number of sequenced transcripts confirming both adjacent splice sites. To account for the higher quality of full-length cDNAs, we counted them twice. Based on these confirmation counts, we compiled a high-confidence sequence-confirmed splicing (SCS) data set as follows. As positive examples, it contains 762 IR and 173 ES events for which each isoform was found in at least two sequenced transcripts. To obtain a ratio between alternative and constitutive splicing that reflects current knowledge about the prevalence of AS in the *A. thaliana *genome [2,31], we included 14,492 constitutive exons and 13,132 constitutively spliced introns. These negative examples were sampled from exons and introns with confirmation counts greater or equal to 5, for which no alternative transcript had been sequenced. On average, constitutive exons and introns in the SCS set are confirmed by 15 and 19 sequenced transcripts, respectively. The composition of the exon and intron SCS data sets is available as supplemental material from Additional File 1 and 2, respectively.

### A supervised two-stage approach for the identification of AS

**Figure 1:**
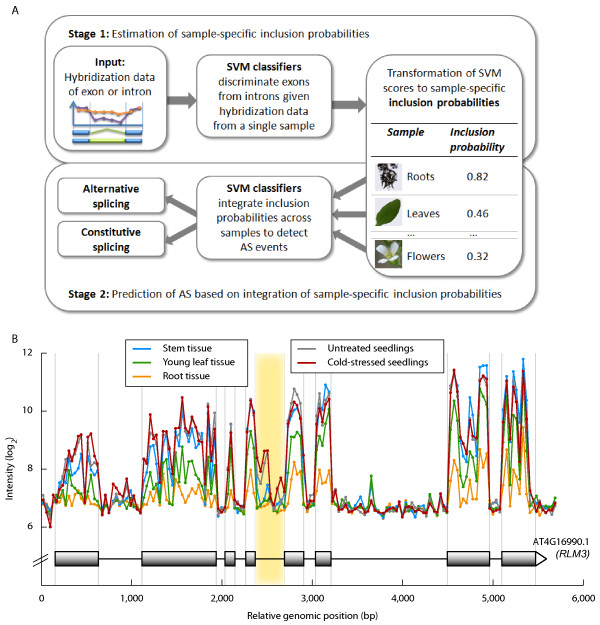
**Workflow of the AS detection method and exemplary gene with observed hybridization profile**. (A) Workflow of the supervised two-stage AS detection method. In the first stage, SVMs are employed to classify a given gene segment as an exon or intron, based on the hybridization signal observed in a single sample. The resulting SVM scores are transformed to probabilistic confidences to estimate the probability that this segment is included in mature mRNA transcripts. In the second stage, these inclusion probabilities are combined across samples by another SVM classifier to detect alternative splicing. (B) Example gene with hybridization data. The figure illustrates the annotated structure and measured hybridization data for the anti-fungal resistance gene RLM3 (AT4G16990), which was among the top 1% in our genome-wide screen. The logarithmized probe intensities for different samples are plotted against the relative genomic positions of the probes. The highlighted intron shows a characteristic hybridization pattern for differential alternative splicing.

In this work, we developed a supervised two-stage approach for the detection of IR and ES events from tiling array data (Figure 1). At the core of the first stage, a set of support vector machine (SVM) classifiers distinguish exons from introns, based on features derived from their hybridization patterns and their relative position in the spliced transcript. We employed these exon-intron classifiers to decide for each tissue or stress treatment in isolation whether a given mRNA segment (i.e., an exon or intron) is included or excluded in a mature mRNA transcript under this condition. This step is based on the assumption, that all included mRNA segments (i.e., constitutive exons and retained introns) show similar hybridization patterns, which can accurately be distinguished from those of excluded mRNA segments (i.e., constitutive introns and skipped exons). Subsequently, the resultant SVM scores were transformed into probabilistic confidences indicating the inclusion probability of a given exon or intron in the mature mRNA transcripts of a gene for a given condition.

Clearly, the difficulty of the exon-intron classification task, solved in the first stage, varies from gene to gene depending on the expression level, as the difference between the hybridization signals of intronic and exonic probes increases with gene expression. To alleviate the expression-dependent differences of exonic and intronic signal levels, we employed a meta-classifier, consisting of *M *= 10 SVM classifiers, each of them specialized to a certain range of expression values. These ten SVM classifiers were independently trained on hybridization patterns corresponding to exons and introns not included in the SCS data set and posterior class probabilities estimated (see Methods). We verified that this meta-classifier consisting of several expression-specific SVM classifiers indeed achieved higher accuracy than a single SVM classifier (Additional File 3).

In the second stage, another layer of classifiers integrates these single-sample inclusion probabilities across multiple hybridization samples to predict alternatively spliced segments. We trained different SVM classifiers for the prediction of different types of AS. One of these classifiers learned to infer ES events from untypically low exon inclusion probabilities; another one was trained to detect IR events from untypically high inclusion probabilities of introns. In addition to the sample-specific inclusion probabilities, we provided these predictors of AS with sample-matched gene expression values allowing them to re-weight the inclusion probabilities in an expression-dependent manner.

The proposed two-layer architecture allows to optimally use the existing labeled data: abundant constitutive splicing events are used to train the model dealing with highly variable the hybridization intensities to obtain stable exon/intron inclusion rates, while the much fewer AS events are used to predict AS based on the inclusion estimates.

### Prediction accuracy assessed in comparison to genome annotation and sequence data

**Figure 2:**
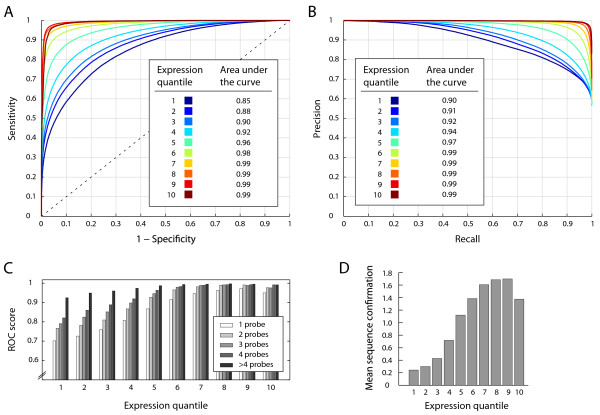
**Performance of the exon-intron classifier**. (A) Accuracy as measured by the area under the ROC curve for different gene expression quantiles. (B) Classification accuracy assessed with precision-recall curves. (C) Prediction accuracy as a function of gene expression levels and the number of probes interrogating an exon/intron. (D) Histogram showing correlation between sequence confirmation and expression level of mRNA transcripts.

**Figure 3:**
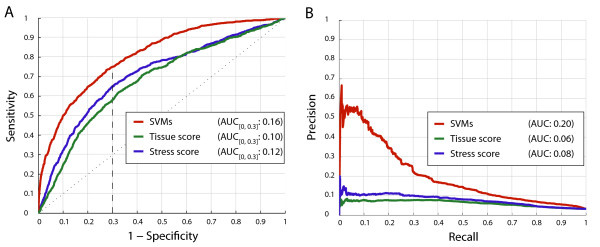
**Performance of predictors for alternative and differential splicing**. The classification accuracy of the SVM-based predictor was compared to unsupervised predictors for the detection of tissue-specific and stress-dependent differential AS events, respectively. (A) Depicted are ROC curves assessed on the unified exon and intron SCS sets. The area under the curve was computed for the x-axis interval corresponding to a false positive rate between 0 and 0.3. (B) Shown is the corresponding Precision-recall curve.

In order to assess the prediction accuracy of the exon-intron classifiers, applied in the first stage of our method, we performed a 5-fold cross-validation on a large set of 71,928 constitutive exons and 47,952 constitutive introns, obtained from the TAIR annotation [27]. For this purpose, we employed receiver operating characteristic (ROC) as well as precision-recall analysis. Depending on gene expression levels, exon-intron classifiers achieved values for the area under the ROC curve (auROC) ranging from 0.85 to 0.99 indicating very high accuracy (Figure 2A). Precision-recall plots further confirmed that exons can be distinguished from introns with very high recall rates at a low false discovery rate (Figure 2B). In addition to gene expression level, as measured on tiling arrays, two more factors were identified to influence classification accuracy. Sequence confirmation had a positive effect, partly, because it is also strongly correlated with gene expression level, but probably also because label uncertainty decreases as sequence confirmation increases (Figure 2D). Furthermore, the number of tiling probes interrogating an exon or intron also impacts prediction performance. Accuracy was found to increase with the number of informative probes, especially for genes expressed at low levels (Figure 2C). The accuracy values shown here are the result of carefully selecting features on the basis of their discriminatory power (see Additional File 4). Whereas a reliable benchmark set for exon/intron classification could readily be obtained from the annotation, assessing the accuracy of AS predictions resulting from the second-stage classifiers was more challenging. Here we used the EST/cDNA-based SCS data set (see above) to evaluate our predictions of AS in a 5-fold cross-validation. ROC plots show a large overlap between our predictions and the SCS data (Figure 3), notwithstanding the fact that there are caveats to the direct comparison between array-based and sequence-confirmed AS events. Importantly, tissues and conditions analyzed with tiling arrays are not matched to those sampled by ESTs and cDNA sequencing, and due to inconsistent labeling of EST origin, it is hardly feasible to filter EST data for a more direct comparison to tiling array data. The lack of deep EST and cDNA data for some of the conditions represented in the tiling array data sets analyzed here implies that cases which are labeled as constitutively spliced in the SCS data set, may potentially be true examples of alternative splicing if a broader set of conditions is considered. Effectively, this can result in exaggerated estimates of the false-discovery rate of tiling array-based predictions of AS as benchmarked on the SCS data set. Similarly, such a comparison likely underestimates the true recall rate of our AS predictions. Nonetheless, the strong enrichment of our predictions with known cases of AS directly confirms the validity of many of these predictions and allows us to estimate an upper bound of the false discovery rate.

### A comparison of supervised and unsupervised AS detection methods

Although it is difficult to estimate absolute values for the accuracy of tiling array-based predictions of AS using the SCS data set as a benchmark, it is, however, useful to compare the predictions of different methods to each other in terms of their relative enrichment with known cases of AS contained in the SCS data set.

Since most of the previous array-based approaches for profiling or discovery of AS events [21,22,32] are restricted to other types of microarray platforms, such as exon and/or exon junction arrays, it was not possible to directly compare our method to these approaches.

Here, we selected two representative tiling array-based approaches for the method comparison; an approach by Ner-Gaon *et al*. [24], which had to be modified to enable a direct comparison to our method, and another ANOVA-based test, similar to MIDAS http://www.affymetrix.com/support/technical/whitepapers/exon_alt_transcript_analysis_whitepaper.pdf. Both methods are unsupervised, relying on statistical testing. In essence, the former approach identifies potentially retained introns for which the mean probe signal is significantly higher than the mean signal of other introns of the same gene and statistically similar to the mean exonic signal. Similarly, the naive ANOVA-based approach directly takes the hybridization signals as input. First the overall gene expression levels are normalized to correct for expression differences across experimental conditions. Subsequently, the statistical test identifies differentially spliced introns, based on the assumption that their normalized probe signal levels significantly differ between the analyzed samples.

**Figure 4:**
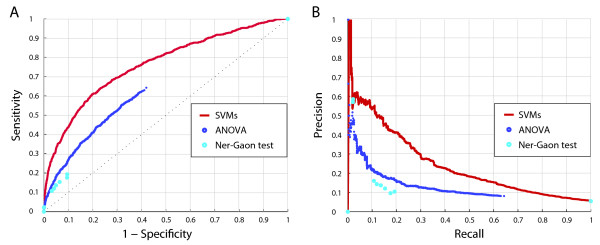
**Performance of supervised SVM-based method vs. unsupervised statistical methods**. The SVM-based approach was compared to unsupervised methods based on statistical tests. The prediction accuracy was assessed on the SCS data set of constitutively and alternatively spliced introns confirmed by EST/cDNA data (see main text). (A) ROC curves. (B) Precision-recall curves.

We compared the three different array-based methods for the identification of IR events relative to the SCS data set by means of ROC analysis. We found that IRs predicted by our supervised learning approach are significantly stronger enriched for sequence-confirmed AS events than the results of the two other AS detection methods that are based on statistical tests (Figure 4A). Particularly, precision-recall curves show that both statistical testing methods achieve considerably lower agreement with known AS events (Figure 4B).

Moreover, we evaluated to what extent different design choices (i.e., different SVM kernels) and tiling array-derived features contributed to the accuracy of our method and how it compares to simpler ad-hoc procedures for the inference AS events (Additional File 5).

### Genome-wide identification of AS

Using the SVM-based predictors of AS, we conducted a whole-genome analysis of all introns contained in the top 50% of TAIR annotated genes with highest expression level. We tested a total of 53,669 internal exons and 68,006 introns, contained in 9,745 and 11,528 TAIR annotated genes, respectively.

**Figure 5:**
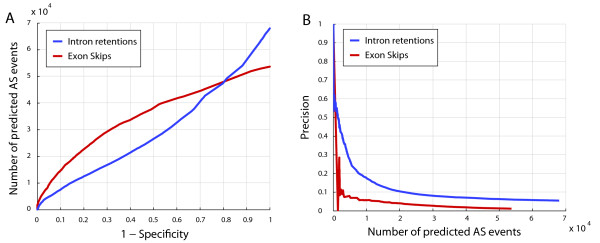
**Cumulative performance of the whole-genome screen**. Number of genome-wide array-based ES and IR predictions as a function of (A) false positive rate (FPR) and (B) precision (PPV). FPR and PPV were assessed on the SCS set (see main text), depending on the prediction score cutoff used for class discrimination.

To quantify the uncertainty associated with each prediction, SVM outputs were transformed to probabilistic confidences using the SCS data sets as a reference (see Figure 5). Taking a stringent cutoff, conservative genome-wide predictions included 1,355 IR events.

We also performed a whole-genome screen for exon skips, which resulted in the prediction of 1,839 candidates that are expected to be enriched with exon skipping events. However, the predicted ES events are considered as unreliable, as the observed overlap with the SCS data set is substantially lower. The IR and ES events predicted genome-wide are available as supplemental material in Additional Files 6 and 7. The confidence scores computed for all introns and exons considered in our genome-wide study of AS can be found in Additional File 8.

### External validation against AS events derived from RNA-seq data

**Figure 6:**
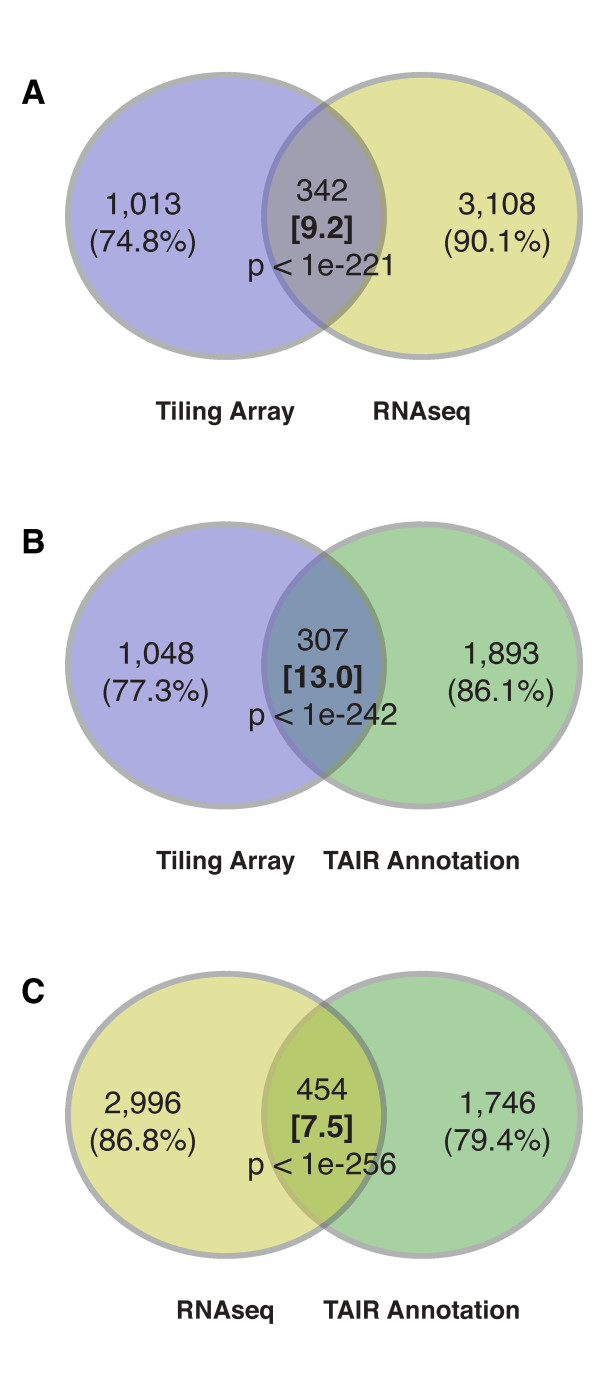
**Overlap of array-based predictions with RNA-seq data and the TAIR annotation**. Overlaps between tiling array-based IR predictions, and IR events derived from RNA-seq data and present in the TAIR annotation illustrated as Venn diagrams. Values in brackets indicate overrepresentation compared to random. Overlap significance was assessed using a hypergeometric test. (A) Overlap between tiling array-based IR predictions and TAIR annotation. (B) Overlap between RNA-seq-derived IR events and TAIR annotation. (C) Overlap between tiling array-based predictions and RNA-seq-derived IR events.

We performed an additional external validation of our genome-wide predictions of IR by comparing them to a recently published RNA-seq data set [14]. From the RNA-seq read data, which covers diverse abiotic stress conditions, we derived IR events (see Methods) and determined the overlap with tiling array-based predictions. More than 25% of our predictions are also supported by the RNA-seq data - a > 9-fold enrichment over random (Figure 6A). We note, however, that annotated AS events are more strongly overrepresented among our predictions (13-fold enrichment) (Figure 6B) and furthermore that the overrepresentation of annotated AS events among those derived from RNA-seq data is weaker (7.5-fold) (Figure 6C). Thus, tiling array-based inference of AS recovered annotated IR events more accurately than could be achieved with a comprehensive RNA-seq data set [14]. This result can in part be explained by the fact that the tiling array data set covers a richer set of experimental conditions than the RNA-seq data set. In total, 525 out of our 1,355 genome-wide predictions of IR (almost 40%) are supported by either annotation or RNA-seq data with 124 events being present in all three sets. We expect that many more of our predicted AS events will be confirmed by more comprehensive sequence experiments.

### Tissue-regulated and stress-induced AS

Inferring AS based on tiling arrays rather than EST and cDNA sequences has the advantage that complete information for all analyzed samples is available. We thus investigated splicing profiles across tissues and stress conditions for the set of genome-wide predicted AS events. To detect tissue-specific and stress-induced AS events, respectively, we implemented two scores integrating multiple samples on top of the single-sample inclusion probabilities, which were estimated in the first stage of our method. Tissue-regulated AS was inferred from high differences of tissue-specific inclusion levels. Stress-regulated events were identified based on varying inclusion levels between stress treatments and controls. Accordingly, we defined tissue and stress scores (see Methods section for details) and selected differentially spliced introns using a stringent cutoff.

We identified 478 IR events showing tissue-specific regulation. 244 IR events were found to be differentially included in mRNA transcripts between stress treatments and the corresponding controls. For 139 IR events we observed both tissue-specific and stress-dependent AS. The predicted AS events listed in Additional Files 6 and 7 are specifically marked to indicate tissue-specificity and/or stress-dependency.

## Discussion

### Tiling arrays are well-suited to study AS in a plant model

Many of the commonly known biases and limitations inherent in sequence-based approaches for the study of AS are ameliorated by tiling arrays. EST coverage usually increases toward the 3' and 5' ends of transcripts as a consequence of over-representation of end-sequence reads in the respective libraries, and similar biases resulting from oligo(dT)-based priming are commonly known for cDNAs [24]. Furthermore, traditional sequence-based approaches with limited sequencing depth inevitably result in a poor coverage of genes with low expression. Consequently, AS events occurring in genes with expression restricted temporally, spatially, or to certain environmental conditions are often missing or underrepresented in current databases. However, tiling array data exist and are publicly available for a large variety of tissues, developmental stages, and environmental conditions for many model organisms including *A. thaliana *[33]. Tiling arrays provide a well-suited platform for profiling AS in plants, not least because in contrast to other AS-sensitive arrays, only tiling arrays allow for the discovery of novel intron retention events. A better understanding of this most prevalent type of AS in plants will also contribute to the elucidation of the mechanistic differences in splicing between plant and animal systems.

### Accuracy of the proposed method

With the amount of training data available for *A. thaliana*, SVMs were found to more accurately recover known IR events than the unsupervised statistical methods considered in our comparison. A possible explanation for this is the robustness of SVMs against high levels of noise as observed in the microarray data. Furthermore, statistical tests often rely on modeling assumptions (e.g., Gaussian distributions) which might not necessarily be true for real microarray data. In contrast to statistical methods which are normally exclusively based on normalized probe intensities, our SVM-based classifiers incorporate additional features, which were found to increase the classification accuracy (Additional File 4).

We observed much lower accuracy for the ES classifier compared to IR predictions. This may be a consequence of ES events being less frequent in *A. thaliana *than IR events (173 confirmed ES events vs. 762 confirmed IR events in the SCS data set). Hence also the class distribution, i.e., the ratio between constitutively and alternatively spliced exons/introns, is much more imbalanced for ES events than for IR events (1 : 84 and 1 : 17 respectively), which makes the ES classification task more difficult.

### Applicability of the proposed method

The supervised AS detection method proposed here is applicable to other organisms, provided that sufficient training data, i.e., EST/cDNA sequences, are available from genome annotations and sequence databases. Already at moderate sequencing depth, ESTs and cDNAs typically confirm sufficiently many exons and introns to derive ample training data for the exon-intron classification in the first step of the algorithm, and hence this should be applicable to a wide range of non-model organisms. The second step of our algorithm, which involves the training of SVM classifiers on known AS events, depends much more on comprehensive annotations. However, a statistical test could replace the supervised approach here, and this slightly modified strategy would also be applicable to species that are poorly annotated with respect to AS. Conceptually, our method could also be applied to focused array designs provided that intron probe sets are available. If exon-exon junction probes were integrated on the employed array platform, the respective hybridization signals could be incorporated as additional connectivity features supporting the condition-dependent inclusion or exclusion of an exon in mature mRNA transcripts. Analogously, exon-intron junction probes could be utilized for the detection of retained introns. However, the analysis of exon arrays is not directly possible, because our method requires intronic signal levels for the estimation of single-sample inclusion probabilities.

### Validation of tiling array-based AS predictions relative to EST/cDNA sequences, RNA-seq data, and the genome annotation

As most studies of AS have the goal to discover new AS events for which by definition no sequence-evidence exists, it has become a common practice to independently validate predictions by reverse-transcriptase PCR (RT-PCR) experiments [24,34]. This validation method provides an accurate means for assessing the confidence in predicted, as yet unknown AS events, but is typically only used to confirm a few dozen cases. Instead of performing biological validation experiments, we adopted an alternative strategy of evaluating our predictions on a set of known, sequence-confirmed AS events, derived from large collections of publicly available transcript sequences. Such a comparison can easily be based on thousands of cases, but there are caveats to the interpretation of the results. First, the number of AS events contained in our SCS test set is constrained by the number of available EST and cDNA sequences, which mostly cover highly expressed genes. Therefore, our evaluation set is biased toward high expression levels, which limits the generalizability of the results for genes expressed at low levels. Second, as the TAIR annotation as well as sequence databases are incomplete with respect to AS at present, an unknown number of constitutively labeled segments in our test set may actually undergo AS, and these mislabelings may distort evaluation results, particularly the estimation of false positives. To partially overcome these limitations, we complemented this first evaluation strategy by a comparison against ultra-high throughput sequencing data. These data were generated by Filichkin *et al*., who recently published a study of AS, for which they profiled several *A. thaliana *tissues and diverse stress conditions [14] using RNA-seq. The comparison of AS events inferred from tiling arrays to those independently derived from RNA-seq data showed a highly significant overlap between both sets of results (9.2-fold overrepresentation, p-value < 10^-221^) (Figure 6). Interestingly, even though RNA-seq is already replacing splicing-sensitive microarrays as the method of choice for studying AS [35,36,14], the RNA-seq data presently available for *A. thaliana *does not appear to more accurately reflect annotated cases of AS (Figure 6). Although these data set comparisons do not allow us to accurately estimate the performance of our tiling array-based inference, the large number of predictions supported by either RNA-seq-derived or annotated IR events (almost 40%) and the considerably smaller relative overlap between these two sets makes it plausible that many more of our predictions are valid (Figure 6) and that the extent of AS is still likely to be underestimated.

### A catalog of newly identified AS events will enable future research

The compilation of array-based predictions generated in this study adds to our current knowledge of the prevalence of AS in *A. thaliana *[27,2,37] and provides new insights into tissue- and stress-specific regulation of AS. The fact that our predictions were made with respect to a large, but well-defined panel of plant organs, developmental stages, and stress treatments is an advantage over sequence-based AS databases, for which sample origin information is typically difficult to map. Since we studied the tissue-dependent occurrence of isoforms, our work constitutes a starting point for functional characterization as well as for studying regulation of differential alternative splicing. The latter task could be approached based on correlation of expression patterns of known splicing regulators with the putatively targeted, "co-spliced" exons and introns, showing consistent splicing profiles across tissues. Furthermore, putative splicing factor binding sites could be detected, based on a search for overrepresented motives in the flanking sequences of co-spliced exons or introns. [8,38]. Finally, a splicing-regulatory network integrating the predicted relationships between splicing factors and target exons and introns could be inferred for *A. thaliana*. To provide a basis for studying the mechanisms governing AS regulation and its physiological implications on a systems level, future research focusing on the elucidation of the regulatory interactions between *trans*-acting splicing factors and their *cis*-acting pre-mRNA motives appears promising.

## Conclusions

In this paper we describe a supervised machine learning-based method for large-scale detection and profiling of alternative splicing from a quantitative tiling array platform.

While limited amounts of known AS events are available, which serve as labeled training data for supervised AS detection methods, the number of reliably annotated constitutive exons and introns is very large. We therefore designed a two-stage classification procedure which first learned to discriminate constitutive exons and introns in single samples and subsequently integrated scores across samples to obtain predictions of AS. Specifically, we trained ten SVMs in the first stage, which were specialized to appropriate ranges of gene expression, and discriminated exons from introns, based on diverse features derived from the corresponding hybridization pattern and position in the transcript. The predicted SVM scores were in turn transformed to probabilistic confidences which served as an estimator for the probability that a given exon or intron is contained in the mRNA of a gene expressed under a particular environmental condition. In the second step the single-sample inclusion probabilities were combined across samples for each exon and intron. The resulting all-sample score proved to be an appropriate means for the discrimination of constitutively and alternatively spliced segments and was found to be more accurate than the outcome of statistical tests when benchmarked against known AS events. We thus applied the all-sample SVM-based prediction score in a genome-wide screen to discover novel IR events. Comparisons to a recently published comprehensive RNA-seq data set [14] and the latest genome annotation directly validated almost 40% of our genome-wide predictions and suggest that our method re-discovered AS events present in these benchmarking sets with an accuracy that is comparable to that of the presently available RNA-seq data.

## Methods

### Definition of ES and IR

As skipped exons we considered exons which are present in at least one transcript, while in at least one other transcript the same region is entirely contained in an intron. Additionally, we required that these two transcripts have at least one upstream exon and one downstream exon in common. As retained introns we treated introns which are spliced out from at least one isoform, while at least one additional isoform exists in which an exon spans the same region.

### Inferring AS events from EST/cDNA sequences

The gene models used in this computational analyses of AS incorporate TAIR annotated transcripts, as well as EST/cDNA sequence information. We obtained full-length cDNA sequences from RIKEN [28] and additionally collected EST sequences from dbEST [39] (as of November, 15, 2007). At first we built a cDNA/EST-based gene structure using BLAT [17] to align EST/cDNA sequences to the genome of *A. thaliana*. For detailed information about the pipeline used for generating the EST/cDNA-based gene structure we refer to Sonnenburg *et al*. [40]. We generated a second gene structure, which was parsed from a gff3-file containing the TAIR7 annotation [27]. The annotation-based gene structure was in turn combined with the EST/cDNA-based gene structure by merging overlapping transcripts located on the same strand. Finally, we built splicing graphs for each gene in which exons correspond to nodes with genomic coordinates and introns to joining edges between exons. AS was then inferred based on splicing graphs according to the definitions given above.

### Generation of the SCS data set

In order to collect positive examples for the SCS set (see Table 1), we detected IR or ES events based on splicing graphs and included them in the SCS set, if both isoforms were confirmed by at least two EST/cDNA sequences. For the negative examples in the SCS set, which correspond to constitutive exons and introns, respectively, we required at least 5-fold EST/cDNA confirmation for each of the two adjacent splice sites. Since the high-quality cDNAs provide more reliable evidence for the existence of splice sites than EST sequences, we counted them twice.

**Table 1:** Data sets used for SVM training and performance evaluation.

Data set	Positive examples	Negative examples
Constitutive types of segment set	71928 annotated constitutive exons	47952 annotated constitutive introns

intron SCS set	762 IR events with EST/cDNA evidence for both splice forms	13132 sequence-confirmed constitutive introns

exon SCS set	173 ES events with EST/cDNA evidence for both splice forms	14492 sequence-confirmed constitutive exons

The table itemizes all data sets on which the classification performance of our methods was assessed, and specifies the positive and negative examples used for SVM training and evaluation.

In order to evaluate our method we compiled a representative SCS set, adapting the class distribution to the ratio *r_AS _*of alternatively spliced genes we would expect for the whole genome, based on published surveys of AS. We set *r_AS _*= 30%, using an overestimation of an EST/cDNA-based estimate by Wang *et al*. [2] who found that 22% of the genes in the *A. thaliana *genome undergo alternative splicing. Due to the limited amount of sequence data used in this study, the estimate reported by Wang *et al*. is likely to be an underestimation of the true ratio [3]. This view is also supported by the work of Simpson et al., who reported that AS is estimated to occur in a third of the genes in *Arabidopsis *[31]. We thus increased the ratio computed by Wang and Brendel to 30%, which is closer to current expectations about the prevalence of AS in *A. thaliana*.

Based on a list of genes undergoing sequence-confirmed AS events taken from the ASIP database http://www.plantgdb.org/ASIP/Download/, we determined the proportion of genes undergoing IR or ES events among all alternatively spliced genes which is *r_IE _*= 70; 9%. Combining the two estimated ratios, we computed the proportion of genes undergoing IR or ES events among all genes: *r *= *r_IE _r_AS _*= 21; 3%. The number of genes with a single isoform was chosen, such that this ratio *r *is reflected by the SCS set.

Since we evaluated the considered AS detection methods on a set of introns and exons, respectively, we had to infer the class distribution on the segment level from the ratio *r *determined on the gene level. To this end, we first determined the number intron retentions *i_a _*= 996 and exon skips *e_a _*= 259, which occurred in *g_a _*= 964 alternatively spliced genes, based on splicing graphs built from the EST/cDNA data. Given the number of alternatively spliced genes *g_a _*= (*g_a _*+ *g_c_*) ·*r*, we computed the number of single-isoform genes $g_{c}=g_{a}\cdot \frac{1-r}{r}=3,568$. Based on a statistical analysis by Reddy, who observed that the average gene of *A. thaliana *is composed of 5 exons and 4 introns [3], we calculated the number of constitutively spliced exons *e_c _*= 5 · *g_c _*+ 4 · *g_a _*= 21, 696 and the number of constitutively spliced introns *i_c _*= 4 · *g_c _*+ 3 · *g_a _*= 17, 164 under the simplifying assumption, that there is only one AS event per alternatively spliced gene. Our approximation resulted in a class distribution of *i_a _*: *i_c _*= 1 : 17 for the intron SCS set and *e_a _*: *e_c _*= 1 : 84 for the exon SCS set (see Table 1). As the number of available constitutive introns and exons with high EST/cDNA evidence was insufficient for adjusting the respective SCS sets to the predetermined class distributions, the corresponding segments were sampled with replacement from a basic population.

### Array design

The expression data was measured by the Affymetrix GeneChip Arabidopsis Tiling 1.0R Array, which comprises more than 3.2 million perfect match and as many mismatch probes tiled throughout the whole non-repetitive portion of the *A. thaliana *genome. The central positions of adjacent 25-mer probes are spaced 35 base pairs on average, leaving a gap of approximately ten base pairs between the probes.

### Experimental conditions of the hybridization samples

The analyzed dataset comprises signal levels measured in 11 tissues and developmental stages of the *A. thaliana *Col-0 referenced strain [41], as well as hybridization data of 13 abiotic stress treatments [13]. The environmental stress response data were derived from plant seedlings, which were exposed to diverse stress conditions at preassigned time points. As three biological replicates were available for each hybridization sample, the whole dataset comprehends 72 tiling array experiments. For detailed information about the plant material, growth conditions, probe preparation, and array hybridization, the reader is referred to [41,13].

### Normalization of the tiling array data

In order to compensate for background noise, cross-hybridization, and probe-specific effects, we used diverse normalization techniques. We corrected for the uneven hybridization background of individual microarrays using a mean image subtraction technique [42]. To correct for the inherent variation of the laboratory experiments, which causes differing intensity distributions between arrays, the distributions of probe intensities were mapped to the mean of the empirical intensity distributions of all arrays by quantile normalization [43]. Since the whole sequence of the Arabidopsis genome was known a priori [44], we could address the problem of cross-hybridization by identifying repetitive k-mers in the genome and by excluding all repetitive probes from further analysis as described previously [45]. Probe-specific effects were alleviated by using a transcript normalization technique [46].

### SVM-based approach for the detection of AS

We formulated the problem of identifying IR and ES events as a supervised learning task and designed a two-stage classification procedure. First, we trained SVMs to discriminate constitutive exons and introns in single samples. In a second step, the single-sample scores computed in the first step were integrated across samples to predict of IR and ES events.

#### Expression-dependent partitioning of the training data

As gene expression may differ dramatically between genes and experimental conditions, it is difficult to accurately define a global threshold, separating the likewise differing intronic and exonic probe signal levels. Intuitively, it would be more appropriate to choose such a threshold depending on gene expression. This could be achieved by discretizing the complete range of gene expression levels into a fixed number of intervals and defining a local threshold for each such interval. Based on these considerations, we split the training data of the SVMs applied in Stage 1 (*see constitutive segment set *in Table 1) into ten disjoint *expression bins *and then trained ten SVM classifiers, each applicable for a well-defined range of gene expression. The limits of the ten expression bins were defined as the percentiles (*P*_10, _*P*_20, _..., *P*_90_) calculated from the distribution of the median exonic probe intensities measured under each condition for each gene.

#### Extraction of hybridization-based features for exon/intron classification

We trained SVMs on three types of features extracted from the signal levels and positions of the probes, complementary to a given exon/intron *s *and measured in sample *t*: absolute intensity features, *F_abs_*(*s*, *t*), relative intensity features *F_rel_*(*s*, *t*) and positional features *F_pos_*(*s*). The absolute intensity features are given by the vector *F_abs_*(*s*, *t*) = (*P*_20_, *P*_40_, *P*_50_, *P*_60_, *P*_80_) composed of five local intensity percentiles. These percentiles provide a compact representation of the intensity distribution measured in sample *t *by the probes complementary to segment *s*. As each experiment was performed in triplicate, three measurements per probe were included in the calculation.

The relative intensity features *F_rel_*(*s*, *t*) measure the relative expression level of an exon/intron *s *under the experimental condition *t *compared to the whole spectrum of observed probe intensities. These features are not correlated to the absolute expression features and correspond to histograms, built from the probe intensities measured for a certain exon/intron. The discrete intervals, i.e., bins, are defined based on *N *= 5 global percentiles *L *= (*P*_20_, *P*_40_, *P*_50_, *P*_60_, *P*_80_) computed from the intensity distribution of all genic probes in all samples combined. As done for the local percentiles, probe intensities were pooled across replicates. Given the intensity vector representation *I*(*s*, *t*) = (*I*_*t*1_(*p*_1_),..., *I*_*t*3_(*p*_*n*_)) of a segment/sample pair (*s*, *t*), which is measured in triplicate by the complementary probes *p*_1_,..., *p*_*n*_, we calculated the components of *F*_*re*l_(*s*, *t*) by linear interpolation between the global percentiles *L*_1_,..., *L*_*N *_. We first initialized *F*_*re*l_(*s*, *t*) = (0,..., 0), where each component corresponds to one of the *N *= 5 global percentiles. Next, we iteratively assigned each intensity value, i.e., component of *I*(*s*, *t*), to an interval, limited by either the first, the last, or two neighboring global percentiles. If the intensity value was outlying one of the outer global percentiles *L*_1 _= *P*_20 _and *L*_*N *_= *P*_80_, either the first or the last component of *F*_*re*l_(*s*, *t*) was increased by 1. Otherwise, if the intensity value was assigned to an interval limited by two global percentiles, the two corresponding components of *F*_*re*l_(*s*, *t*)*_i _*were increased in a weighted manner. We defined this increase as the reciprocal of the relative distance of the intensity value to the corresponding distance limit. Formally, the *i*-th component *F*_*re*l_(*s*, *t*)*_i _*of the feature vector *F*_*re*l_(*s*, *t*) is defined as:

(1) $$F_{rel}{\left(s,t\right)}_{i}=\sum_{j=1}^{n}\sum_{r=1}^{3}f(I_{tr}(p_{j}),i)$$

(2) $$f(I,i)=\{\begin{array}{ll} 1 & \text{if}\;(I\leq L_{1}\wedge i=1)\vee (I>L_{N}\wedge i=N)\\ \alpha & \text{if}\;L_{k}\leq I<L_{k+1}\wedge i=k\\ 1-\alpha & \text{if}\;L_{k}\leq I<L_{k+1}\wedge i=k+1\\ 0 & \text{otherwise} \end{array}$$

(3) $$\text{where}\;\alpha =\frac{L_{k+1}-I}{L_{k+1}-L_{k}}$$

Since exonic probe signal levels were found to unevenly run across transcripts and often tend to decrease with increasing distance to the 3' transcript end [46], we additionally provided the SVM classifiers with positional features *F_pos_*(*s*) in order to compensate for this bias. These features measure the distances of the probes *p*_1_,..., *p_n_*, complementary to an exon/intron *s*, from the 3' end of a spliced transcript. First, we arbitrarily defined distance limits *L *= (100, 300, 500,..., 1900), each corresponding to a certain number of nucleotides between a probe and the 3' transcript end. Then, we determined these distances for each probe *p*_1_,..., *p_n _*and computed the positional feature vector *F_pos_*(*s*) by linear interpolation of the probe distances between the distance limits *L*, using the same procedure as for the relative intensity features.

#### Training and evaluation of exon/intron classifiers

For the exon/intron classification SVM models with linear kernel were trained for each of the ten expression bins and a 5-fold cross-validation was performed on the *constitutive segment set *(see Table 1) to assess the prediction accuracy. The data were partitioned such that 60% were used for training and 20% for model selection and testing, respectively. In each iteration the optimal value of the soft margin parameter *C *was determined on the validation set by grid search (*C *∈ {0.001, 0.01, 0.1, 1, 10, 100, 1000}). The prediction accuracy was estimated on the test set in terms of auROC and averaged across the 5 folds to produce a single score. For SVM training and classification we used an efficient CPLEX-based implementation http://www.ilog.com/products/cplex/[47].

#### Estimation of single-sample inclusion probabilities

To obtain a score measuring the sample-specific inclusion of exons/introns in mRNA transcripts, we transformed the predicted SVM scores to probabilistic confidences, which are comparable between variably parametrized SVMs trained on different datasets. In essence, the algorithm used for this purpose estimates the conditional likelihood *P*(*y *= 1 | *f_svm_*(*s*, *t*)) that segment *s *is an exon, given the SVM score of segment/sample pair (*s*, *t*) and learns a mapping from SVM outputs to confidences, which is based on monotonically increasing piecewise linear functions. For a detailed description of the algorithm the reader is referred to Sonnenburg *et al*. [40].

#### Detection of AS by integration of single-sample predictions

In the second stage of our classification procedure the single-sample inclusion probabilities were used as features for a second SVM-based model which was in turn applied to produce all-sample scores allowing for the detection of unspecifically regulated AS as well as tissue-regulated or stress-induced differential splicing. Since the array-based prediction of AS requires sufficiently high differences between intronic and exonic probe intensities, we restricted further analyses to the 50% genes with highest expression level. These genes were selected, based on the median signal level of the exonic probes, pooled across conditions and replicates.

The SVM classifiers of Stage 2 were provided with the single-sample inclusion probabilities from Stage 1 and additional expression features. First, each exon/intron was represented by a vector, composed of the inclusion probabilities predicted for each sample. The single-sample scores were sorted in descending order, such that the first and last component correspond to the sample with maximum and minimum inclusion probability, respectively. In a second step, this sorted single-sample score vector was concatenated with an expression feature vector of equal length, which captured the expression level of the flanking exons, given as the median signal level of the complementary probes. The expression feature vector was sorted in consistent order, such that dependencies between inclusion probabilities and gene expression levels could be learned. In order to distinguish retained from constitutive introns, linear SVMs were were trained and evaluated on the intron SCS set (see Table 1) using a 5-fold cross-validation. Analogously, we separately trained SVMs for the detection of ES.

To extract the IR and ES events predicted with highest confidence, we introduced a threshold for the Stage 2 SVM output values, corresponding to an estimated FDR of 0.5 and 0.7 on the intron and exon SCS set, respectively.

#### Detection of tissue-regulated and stress-dependent differential splicing

To further analyze the sets of predicted IR and ES events with respect to tissue-specific and stress-dependent regulation, we implemented a tissue and a stress score on top of the Stage 2 SVM outputs. The scores are based on the assumption that the inclusion of exons/introns is expected to differ across samples in the presence of differential splicing, whereas less variation is expected for basal AS events, characterized by similar isoform ratios in all samples.

(4) $$S_{tissue}(s)=\max_{t}(p(s,t))-\min_{t}(p(s,t))$$

where *p*(*s*, *t*) denotes the inclusion probability of segment *s *in tissue *t*.

(5) $$S_{stress}(s)=\max_{\left(t,c\right)}|p(s,t)-p(s,c)|$$

where *p*(*s*, *t*) and *p*(*s*, *c*) denotes the inclusion probability of segment *s *for stress treatment *t *and the corresponding control *c*, respectively. The final set of predictions was obtained by imposing a cutoff corresponding to a recall of 0.1.

### ANOVA-based IR detection method

The first test method is based on the assumption that retained introns exhibit hybridization intensities which differ across samples, whereas constitutively spliced introns are expected to consistently show low intensities in all samples. We represented each tested segment *s *with complementary probes *p*_1_,..., *p*_*n *_by |*T*| = 24 single-sample intensity vectors *I*_*t*_(*s*) = (*I*_*t*1_(*p*_1_),..., *I*_*t*3_(*p*_*n*_)) where *I*_*tr*_(*p*_*i*_) denotes the signal level of probe *i *in replicate *r *of sample *t*. To account for differential overall gene expression, we computed the median intensity $m_{t}(g(s))=median(I_{t1}(p_{1}^{c}),...,I_{t3}(p_{k}^{c}))$ of the constitutive exonic probes $p_{1}^{c},...,p_{k}^{c}$ covering the gene *g*(*s*) that contains segment *s *for each sample *t *∈ *T *. We alleviated the effects of differential expression by calculating splicing index vectors $S_{t}(s)=\frac{I_{t}(s)}{m_{t}(g(s))}$ for all samples which correspond to groups in the subsequently performed ANOVA test. Based on the p-value *p*(*s*) resulting from the ANOVA test, we distinguished retained introns from constitutive ones.

### IR detection method by Ner-Gaon et al

The approach by [24] applies statistical tests to identify retained introns from tiling arrays, based on untypically high hybridization signals. The main idea of the authors is that retained introns can be discovered based on a comparison of the probe intensities measured in individual introns to the mean exonic signal level of the respective gene.

We reimplemented the method proposed by Ner-Gaon *et al*. in Matlab, maintaining the main concepts of the algorithm, but modifying it in some aspects to enable a comparison to our method. In contrast to Ner-Gaon, we did not require that each tested intron is covered by at least 3 probes, as 60% of the introns in our SCS set do not fulfill this condition. Furthermore, we disregarded the cutoff for the minimal mean exonic and maximal mean intronic transcript signal level proposed by Ner-Gaon, since we observed highly overlapping intensity distributions of exon and intron probes. Since the assignment of a prediction score to all introns in our SCS set was a necessary prerequisite for the subsequent ROC analysis, we omitted the Benjamini-Hochberg correction for multiple testing, which would have excluded genes with insufficient significance from further analysis. As the transcript classes defined by Ner-Gaon *et al*. do not capture all transcripts, we introduced an additional class for the *undifferentiated *transcripts not inclusive to one of the original classes by Ner-Gaon. Furthermore, for the computation of ROC curves, we had to change the numbering of the transcript classes, such that the predicted class index increases with higher confidence of AS. Subsequently, we mapped transcript classes to equivalent intron classes, since the classification performance was assessed on a set of *introns*. To obtain additional points in ROC space, we increased the number of discrete class labels, i.e. intron classes 1-4, by varying the significance level *α *used for the pairwise comparison of group means from 0.01 to 0.5 in 5 logarithmically spaced steps. For each significance level *α *∈ [0.01, 0.5] and intron *s *assigned to intron class *c*(*s*) ∈ {1, 2, 3, 4}, we computed a significance score *N_α _*(*s*) = *c*(*s*) + (1 - *α*). The final intron score $N(s)=\underset{\alpha }{\max}S_{\alpha }(s)$ results from the significance level *α *with maximal score *N_α _*(*s*).

### Detection of IR from deep RNA sequencing (RNA-seq)

For this analysis we used the read data published in [14] (Short Read Archive accessions SRX006192, SRX006681, SRX006682, SRX006692, SRX006704, SRX006690, SRX006688) obtained with an Illumina Genome Analyzer 1G. We aligned the total of 210 million reads with Palmapper [48] against the *A. thaliana *genome (TAIR 9). In total we were able to align 75 million reads, out of which 4.2 million reads lead to a spliced alignment for the best hit. We merged the alignments of all libraries and to increase specificity of the alignments we filtered out those which had more than 1 mismatch or a minimal segment length in spliced alignments that was shorter than 8nt. We then considered every annotated intron in the TAIR 9 annotation and checked whether all of the following conditions were satisfied: (a) the median read coverage in the intron was larger than 2, (b) at least 75% of the intronic positions were covered by at least one read, (c) the mean intron coverage of the intron was at least 10% and at most 120% of the average coverage of the two flanking exons and the average coverage of the two flanking exons differed at most 4-fold. This led to a total of 3, 691 detected IR events out of 125, 921 annotated introns. After removing redundancy, we finally obtained 3, 450 IR events. We have also derived AS events using more stringent filtering settings, but did not observe a significantly increased enrichment when compared with the annotation (data not shown).

### Overlap between tiling array-based, RNA-seq-derived, and annotated IR events

All segments were mapped to the TAIR9 genome release. Comparisons were conducted with respect of all introns annotated in TAIR9 and mapped to the nuclear chromosomes. Statistical significance based on the Hypergeometric test as well as representation factors were calculated using an implementation by Jim Lund http://nemates.org/MA/progs/overlap_stats.html with 125, 921 as the total number of introns.

## Authors' contributions

JE and GZ conceived the method and wrote the manuscript. JE implemented the method in Matlab and performed validation and predictions. SL helped with interpreting the data. GR initiated and supervised the project, implemented the detection of intron retention events from the RNA-seq data and helped writing the manuscript. All authors have read and approved the manuscript.

## Supplementary Material

Additional file 1 **Exon SCS set**. This file lists all constitutively and alternatively spliced exons inferred from EST and cDNA sequences, forming the test set which was used for the validation of our predictions. Along with gene/transcript identifiers, the exon boundary coordinates and the number of sequences confirming adjacent splice sites are stated for each exon. Click here for file

Additional file 2 **Intron SCS set**. Analogous to Additional file 1, this file contains the list of constitutively and alternatively spliced introns, used for the evaluation of our method. Click here for file

Additional file 3 **Classification performance of 1-Bin vs. 10-Bin exon-intron classifier**. We compared the prediction accuracy of two SVM-based classifiers which were trained to distinguish exons from introns: a single SVM classifier, and a meta-classifier which incorporates 10 SVMs, each specialized to a certain range of gene expression levels. The prediction accuracy was assessed on a large evaluation set of annotated constitutive exons and introns. (A) ROC curves. (B) Precision-recall curves. Click here for file

Additional file 4 **Prediction accuracy achieved by different features for exon-intron classification**. This file contains a supplementary table which shows the ROC and precision recall scores achieved by first stage classifiers provided with different combinations of expression features and positional features. Click here for file

Additional file 5 **Classification accuracy of different IR and ES predictors**. This file contains a supplementary table which shows the prediction accuracy assessed for different variants of our AS detection method in terms of ROC and precision-recall scores. Click here for file

Additional file 6 **List of predicted exon skips**. The list itemizes all exons which have been predicted to be differentially included in transcript isoforms with high confidence. For each listed exon the respective ID from the TAIR annotation, as well as the genomic location, and confidence scores for AS are specified. Click here for file

Additional file 7 **List of predicted intron retentions**. This file contains a list of the intron retention events predicted with highest confidence. The corresponding table is structured in the same way as Additional File 6. Click here for file

Additional file 8 **Genome-wide predictions of AS**. This file contains a list of all exons and introns which were analyzed in our genome-wide study of AS. For each listed exon and intron we provide the respective TAIR ID, genomic coordinates, and confidence scores for general AS, as well as tissue-specific, and stress-dependent differential splicing. Click here for file

## References

[B1] Kim H Klein R Majewski J Ott J Estimating rates of alternative splicing in mammals and invertebrates Nat Genet 2004 36 9 915 6 author reply 916-7 10.1038/ng0904-915 15340420

[B2] Wang BB Brendel V Genomewide comparative analysis of alternative splicing in plants Proceedings of the National Academy of Sciences 2006 103 18 7175 7180 http://www.pnas.org/content/103/18/7175.abstract 10.1073/pnas.0602039103 PMC1459036 16632598

[B3] Reddy ASN Alternative splicing of pre-messenger RNAs in plants in the genomic era Annu Rev Plant Biol 2007 58 267 294 10.1146/annurev.arplant.58.032806.103754 17222076

[B4] Black DL Mechanisms of alternative pre-messenger RNA splicing Annu Rev Biochem 2003 72 291 336 10.1146/annurev.biochem.72.121801.161720 12626338

[B5] Johnson JM Castle J Garrett-Engele P Kan Z Loerch PM Armour CD Santos R Schadt EE Stoughton R Shoemaker DD Genome-wide survey of human alternative pre-mRNA splicing with exon junction microarrays Science 2003 302 5653 2141 2144 10.1126/science.1090100 14684825

[B6] Pan Q Shai O Misquitta C Zhang W Saltzman AL Mohammad N Babak T Siu H Hughes TR Morris QD Frey BJ Blencowe BJ Revealing global regulatory features of mammalian alternative splicing using a quantitative microarray platform Mol Cell 2004 16 6 929 941 10.1016/j.molcel.2004.12.004 15610736

[B7] Blencowe BJ Alternative splicing: new insights from global analyses Cell 2006 126 37 47 10.1016/j.cell.2006.06.023 16839875

[B8] Sugnet CW Srinivasan K Clark TA O'Brien G Cline MS Wang H Williams A Kulp D Blume JE Haussler D Ares M Unusual intron conservation near tissue-regulated exons found by splicing microarrays PLoS Comput Biol 2006 2 e4 10.1371/journal.pcbi.0020004 16424921 PMC1331982

[B9] Faustino NA Cooper TA Pre-mRNA splicing and human disease Genes Dev 2003 17 4 419 437 10.1101/gad.1048803 12600935

[B10] Cartegni L Chew SL Krainer AR Listening to silence and understanding nonsense: exonic mutations that affect splicing Nat Rev Genet 2002 3 4 285 298 10.1038/nrg775 11967553

[B11] Garcia-Blanco MA Baraniak AP Lasda EL Alternative splicing in disease and therapy Nat Biotechnol 2004 22 5 535 546 10.1038/nbt964 15122293

[B12] Palusa SG Ali GS Reddy ASN Alternative splicing of pre-mRNAs of Arabidopsis serine/arginine-rich proteins: regulation by hormones and stresses Plant J 2007 49 6 1091 1107 10.1111/j.1365-313X.2006.03020.x 17319848

[B13] Zeller G Henz SR Widmer CK Sachsenberg T Rätsch G Weigel D Laubinger S Stress-induced changes in the Arabidopsis thaliana transcriptome analyzed using whole-genome tiling arrays Plant J 2009 58 6 1068 1082 10.1111/j.1365-313X.2009.03835.x 19222804

[B14] Filichkin SA Priest HD Givan SA Shen R Bryant DW Fox SE Wong WK Mockler TC Genome-wide mapping of alternative splicing in Arabidopsis thaliana Genome Res 2010 20 45 58 10.1101/gr.093302.109 19858364 PMC2798830

[B15] Iida K Seki M Sakurai T Satou M Akiyama K Toyoda T Konagaya A Shinozaki K Genome-wide analysis of alternative pre-mRNA splicing in Arabidopsis thaliana based on full-length cDNA sequences Nucleic Acids Res 2004 32 17 5096 5103 10.1093/nar/gkh845 15452276 PMC521658

[B16] Lazar G Goodman HM The Arabidopsis splicing factor SR1 is regulated by alternative splicing Plant Mol Biol 2000 42 4 571 581 10.1023/A:1006394207479 10809003

[B17] Kent W BLAT-the BLAST-like alignment tool Genome Res 2002 12 4 656 64 11932250 10.1101/gr.229202 PMC187518

[B18] Sasidharan R Agarwal A Rozowsky J Gerstein M An approach to comparing tiling array and high throughput sequencing technologies for genomic transcript mapping BMC Res Notes 2009 2 150 10.1186/1756-0500-2-150 19630981 PMC2764720

[B19] Cuperlovic-Culf M Belacel N Culf AS Ouellette RJ Data analysis of alternative splicing microarrays Drug Discov Today 2006 11 21-22 983 990 10.1016/j.drudis.2006.09.011 17055407

[B20] Okoniewski MJ Yates T Dibben S Miller CJ An annotation infrastructure for the analysis and interpretation of Affymetrix exon array data Genome Biol 2007 8 5 R79 10.1186/gb-2007-8-5-r79 17498294 PMC1929135

[B21] Xing Y Stoilov P Kapur K Han A Jiang H Shen S Black DL Wong WH MADS: A new and improved method for analysis of differential alternative splicing by exon-tiling microarrays RNA 2008 http://rnajournal.cshlp.org/cgi/content/abstract/rna.1070208v1 rna.1070208 10.1261/rna.1070208 PMC2491471 18566192

[B22] Purdom E Simpson KM Robinson MD Conboy JG Lapuk AV Speed T FIRMA: a method for detection of alternative splicing from exon array data Bioinformatics 2008 24 15 1707 1714 10.1093/bioinformatics/btn284 18573797 PMC2638867

[B23] Gaidatzis D Jacobeit K Oakeley EJ Stadler MB Overestimation of alternative splicing caused by variable probe characteristics in exon arrays Nucleic Acids Res 2009 37 16 e107 10.1093/nar/gkp508 19528075 PMC2760813

[B24] Ner-Gaon H Fluhr R Whole-Genome Microarray in Arabidopsis Facilitates Global Analysis of Retained Introns DNA Res 2006 13 3 111 121 10.1093/dnares/dsl003 16980712

[B25] Schölkopf B Smola A Learning with Kernels 2002 MIT Press

[B26] Ben-Hur A Ong CS Sonnenburg S Schölkopf B Rätsch G Support vector machines and kernels for computational biology PLoS Comput Biol 2008 4 10 e1000173. 10.1371/journal.pcbi.1000173 18974822 PMC2547983

[B27] Swarbreck D Wilks C Lamesch P Berardini T Garcia-Hernandez M Foerster H Li D Meyer T Muller R Ploetz L Radenbaugh A Singh S Swing V Tissier C Zhang P Huala E The Arabidopsis Information Resource (TAIR): gene structure and function annotation Nucleic Acids Res 2008 36 D1009 1014 10.1093/nar/gkm965 17986450 PMC2238962

[B28] Sakurai T Satou M Akiyama K Iida K Seki M Kuromori T Ito T Konagaya A Toyoda T Shinozaki K RARGE: a large-scale database of RIKEN Arabidopsis resources ranging from transcriptome to phenome Nucleic Acids Res 2005 33 Database D647 D650 15608280 10.1093/nar/gki014 PMC539968

[B29] Thanaraj TA Stamm S Clark F Riethoven JJ Le Texier V Muilu J ASD: the Alternative Splicing Database Nucl Acids Res 2004 32 suppl_1 D64 69 http://nar.oxfordjournals.org/cgi/content/abstract/32/suppl_1/D64 10.1093/nar/gkh030 14681360 PMC308764

[B30] Noh SJ Lee K Paik H Hur CG TISA: Tissue-specific Alternative Splicing in Human and Mouse Genes DNA Res 2006 13 5 229 243 10.1093/dnares/dsl011 17107969

[B31] Simpson CG Fuller J Maronova M Kalyna M Davidson D McNicol J Barta A Brown JWS Monitoring changes in alternative precursor messenger RNA splicing in multiple gene transcripts Plant J 2008 53 6 1035 1048 10.1111/j.1365-313X.2007.03392.x 18088312

[B32] Anton M Gorostiaga D Guruceaga E Segura V Carmona-Saez P Pascual-Montano A Pio R Montuenga L Rubio A SPACE: an algorithm to predict and quantify alternatively spliced isoforms using microarrays Genome Biology 2008 9 2 R46 http://genomebiology.com/2008/9/2/R46 10.1186/gb-2008-9-2-r46 18312629 PMC2374713

[B33] Barrett T Troup DB Wilhite SE Ledoux P Rudnev D Evangelista C Kim IF Soboleva A Tomashevsky M Marshall KA Phillippy KH Sherman PM Muertter RN Edgar R NCBI GEO: archive for high-throughput functional genomic data Nucleic Acids Res 2009 37 Database D885 D890 10.1093/nar/gkn764 18940857 PMC2686538

[B34] Shai O Morris QD Blencowe BJ Frey BJ Inferring global levels of alternative splicing isoforms using a generative model of microarray data Bioinformatics 2006 22 5 606 613 10.1093/bioinformatics/btk028 16403798

[B35] Pan Q Shai O Lee LJ Frey BJ Blencowe BJ Deep surveying of alternative splicing complexity in the human transcriptome by high-throughput sequencing Nat Genet 2008 40 12 1413 1415 10.1038/ng.259 18978789

[B36] Wang ET Sandberg R Luo S Khrebtukova I Zhang L Mayr C Kingsmore SF Schroth GP Burge CB Alternative isoform regulation in human tissue transcriptomes Nature 2008 456 7221 470 476 10.1038/nature07509 18978772 PMC2593745

[B37] Barbazuk WB Fu Y McGinnis KM Genome-wide analyses of alternative splicing in plants: opportunities and challenges Genome Res 2008 18 9 1381 1392 10.1101/gr.053678.106 18669480

[B38] Desmet FO Hamroun D Lalande M Collod-Béroud G Claustres M Béroud C Human Splicing Finder: an online bioinformatics tool to predict splicing signals Nucleic Acids Res 2009 37 9 e67 10.1093/nar/gkp215 19339519 PMC2685110

[B39] Boguski MS Lowe TM Tolstoshev CM dbEST-database for expressed sequence tags Nat Genet 1993 4 4 332 333 10.1038/ng0893-332 8401577

[B40] Sonnenburg S Schweikert G Philips P Behr J Rätsch G Accurate splice site prediction using support vector machines BMC Bioinformatics 2007 8 Suppl 10 S7 10.1186/1471-2105-8-S10-S7 18269701 PMC2230508

[B41] Laubinger S Zeller G Henz S Sachsenberg T Widmer C Naouar N Vuylsteke M Schölkopf B Rätsch G Weigel D At-TAX: a whole genome tiling array resource for developmental expression analysis and transcript identification in Arabidopsis thaliana Genome Biology 2008 9 7 R112 http://genomebiology.com/2008/9/7/R112 10.1186/gb-2008-9-7-r112 18613972 PMC2530869

[B42] Borevitz J Liang D Plou e D Chang H Zhu T Weigel D Berry C Winzeler E Chory J Large-Scale Identification of Single-Feature Polymorphisms in Complex Genomes Genome Res 2003 13 3 513 523 http://www.genome.org/cgi/content/abstract/13/3/513 10.1101/gr.541303 12618383 PMC430246

[B43] Bolstad B Irizarry R Astrand M Speed T A comparison of normalization methods for high density oligonucleotide array data based on variance and bias Bioinformatics 2003 19 2 185 193 10.1093/bioinformatics/19.2.185 12538238

[B44] Initiative TAG Analysis of the Genome Sequence of the Flowering Plant Arabidopsis thaliana Nature 2000 408 6814 796 815 10.1038/35048692 11130711

[B45] Clark RM Schweikert G Toomajian C Ossowski S Zeller G Shinn P Warthmann N Hu TT Fu G Hinds DA Chen H Frazer KA Huson DH Schölkopf B Nordborg M Rätsch G Ecker JR Weigel D Common sequence polymorphisms shaping genetic diversity in Arabidopsis thaliana Science 2007 317 5836 338 342 10.1126/science.1138632 17641193

[B46] Zeller G Henz SR Laubinger S Weigel D Rätsch G Transcript normalization and segmentation of tiling array data Pac Symp Biocomput 2008 527 538 18229713

[B47] CPLEX Optimization Incorporated Using the CPLEX Callable Library 1994 Incline Village, Nevada

[B48] Jean G Kahles A Sreedharan VT Bona FD Rätsch G RNA-Seq read alignments with PALMapper Curr Protoc Bioinformatics 2010 Chapter 11 Unit 11.6 21154708 10.1002/0471250953.bi1106s32

