# Supplementary figures and images for: Support vector machines-based identification of alternative splicing in Arabidopsis thaliana from whole-genome tiling arrays
Source: BMC Bioinformatics. 2011 Feb 16;12:55. doi: 10.1186/1471-2105-12-55 (PMC3051901; doi:10.1186/1471-2105-12-55)

**A**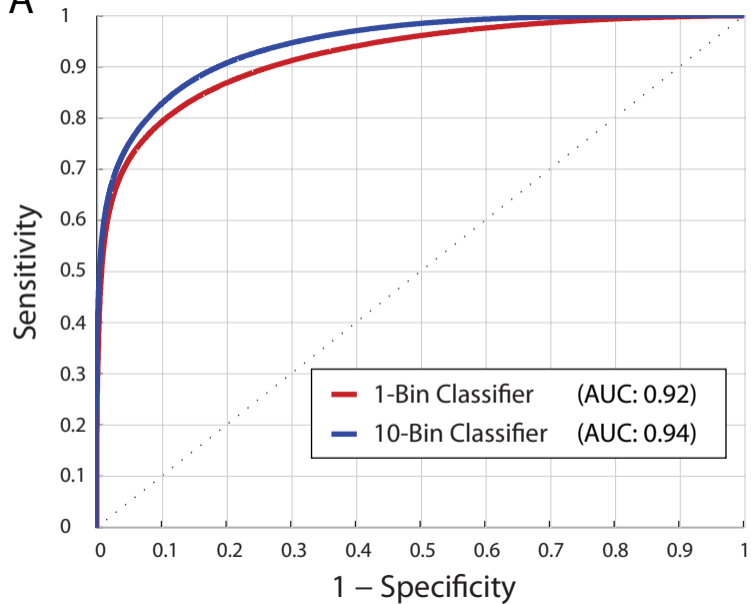**B**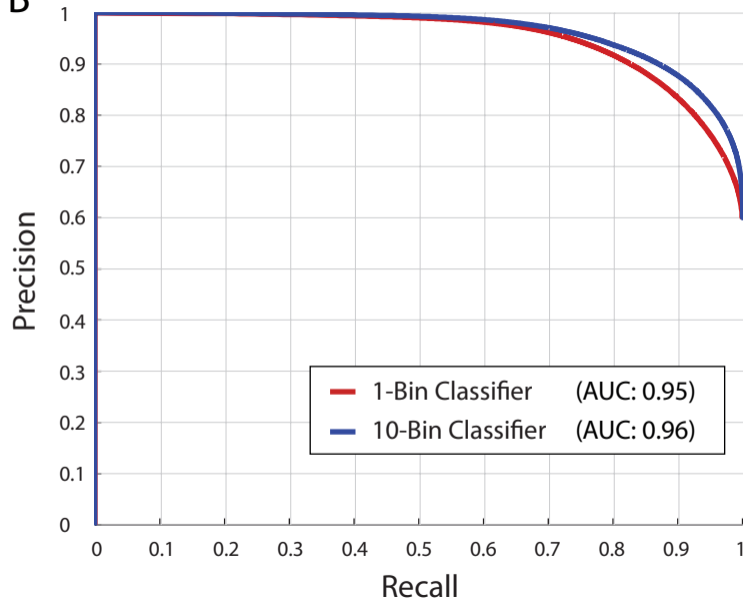

Supplement: Additional file 3 — Classification performance of 1-Bin vs. 10-Bin exon-intron classifier. We compared the prediction accuracy of two SVM-based classifiers which were trained to distinguish exons from introns: a single SVM classifier, and a meta-classifier which incorporates 10 SVMs, each specialized to a certain range of gene expression levels. The prediction accuracy was assessed on a large evaluation set of annotated constitutive exons and introns. (A) ROC curves. (B) Precision-recall curves. [file 1471-2105-12-55-S3.PDF]
