# Supplementary material for: Support vector machines-based identification of alternative splicing in Arabidopsis thaliana from whole-genome tiling arrays
Source: BMC Bioinformatics. 2011 Feb 16;12:55. doi: 10.1186/1471-2105-12-55 (PMC3051901; doi:10.1186/1471-2105-12-55)
Supplement: Additional file 4 — Prediction accuracy achieved by different features for exon-intron classification. This file contains a supplementary table which shows the ROC and precision recall scores achieved by first stage classifiers provided with different combinations of expression features and positional features. [file 1471-2105-12-55-S4.PDF]

Additional File 4 — Prediction accuracy achieved by different features for exon-intron classification.

The table shows the prediction accuracy of the first stage SVM classifiers which were trained on different features to discriminate exons from introns. The performance was assessed on a large set of 71928 constitutive exons and 47952 constitutive introns, known from the TAIR annotation. We computed the area under the ROC (auROC) and precision-recall curve (auPRC) in order to compare SVM-based classifiers trained on different feature types, namely the *absolute intensity features* (AI), *relative intensity features* (RI), *positional features* (P) and *intensity difference features* (ID). The *absolute intensity features* represent the intensity distribution of the probes complementary to a certain exon/intron by means of percentiles. The *relative intensity features* relate the expression level of individual exons/introns to the whole spectrum of intensities measured in constitutive exons and introns. The *positional features* capture the distance of probes to the 3' transcript end, and thereby allow SVMs to correct for an observed bias which finds expression in an uneven intensity distribution across transcripts, i.e., decreasing intensities from the 3' to the 5' transcript end. We also evaluated the contribution of *intensity difference features* to the overall classification performance. This feauture is defined as the difference of the median intensities measured in the two flanking exons of a certain exon or intron.

**Features**	**auROC**	**auPRC**
AI	0.921	0.945
RI	0.934	0.957
AI+RI	0.935	0.957
AI+RI+P	**0.943**	**0.963**
AI+RI+P+ID	**0.943**	**0.963**
