# Supplementary material for: Support vector machines-based identification of alternative splicing in Arabidopsis thaliana from whole-genome tiling arrays
Source: BMC Bioinformatics. 2011 Feb 16;12:55. doi: 10.1186/1471-2105-12-55 (PMC3051901; doi:10.1186/1471-2105-12-55)
Supplement: Additional file 5 — Classification accuracy of different IR and ES predictors. This file contains a supplementary table which shows the prediction accuracy assessed for different variants of our AS detection method in terms of ROC and precision-recall scores. [file 1471-2105-12-55-S5.PDF]

Additional File 5 — Classification accuracy of different IR and ES predictors.

The table shows the classification performance obtained for different all-sample scores, which were used in the second stage of our method to infer AS by integration of the single-sample inclusion probabilities. The performance was assessed on the exon/intron SCS set, based on the area under the ROC (auROC) and precision-recall curve (auPRC), respectively. We compared SVM-based classifiers using different kernels (linear and gaussian) and trained on different feauture types, namely the *single-sample inclusion probabilities* computed in the first stage (IP), *local expression features* (LE), and *global expression features* (GE). The *local expression features* only capture the expression level of the two exons flanking a certain exon/intron, whereas the global ones incorporate the expression of all exons in the respective gene. The accuracies of the SVM-based AS predictors was also compared to those of simple unsupervised classifiers, which infer AS from high differences (*MaxMin-Score*) or high standard deviations (*StDev-Score*) of the single-sample inclusion probabilities. Furthermore, we also evaluated the performance of AS predictors for tissue-specific (*Tissue-Score*) and stress-dependent AS (*Stress-Score*), respectively.

	**Intron Retentions**		**Exon Skips**	
**All-sample Score**	**auROC**	**auPRC**	**auROC**	**auPRC**
SVM-Linear, IP	0.70	0.17	0.63	0.04
SVM-Gaussian, IP	0.70	0.14	0.73	0.04
SVM-Linear, IP+GE	0.71	0.21	0.66	0.03
SVM-Gaussian, IP+GE	0.68	0.17	0.69	0.04
SVM-Linear, IP+LE	**0.75**	**0.24**	**0.73**	0.04
SVM-Gaussian, IP+LE	0.67	0.18	0.72	**0.05**
MaxMin-Score, IP	0.68	0.10	0.71	0.04
StDev-Score, IP	0.68	0.10	0.71	0.04
Tissue-Score, IP	0.65	0.09	0.71	0.03
Stress-Score, IP	0.69	0.10	0.70	0.03
